# Nutrition problems in a severe burn patient with schizophrenia: a case report

**DOI:** 10.11604/pamj.2020.37.257.21269

**Published:** 2020-11-21

**Authors:** Rizka Khairiza, Lucretya Yeniwati Tanuwijaya, Najatullah

**Affiliations:** 1 Division of Plastic Reconstructive and Aesthetic Surgery, Dr. Kariadi Central General Hospital, Semarang, Central Java, Indonesia

**Keywords:** Burn, nutrition, schizophrenia

## Abstract

Hypermetabolic conditions with nutrition deficiencies are common in patients with extensive burns. Balanced nutrition status is required to achieve adequate wound healing. Mental disorder, which tended to make a patient uncooperative, complicates the management. We report the case of a 35-year-old man with schizophrenia who suffered full- and partial-thickness burns in approximately 38% of total body surface area due to attempted suicide. The patient was hospitalized for 66 days and underwent multiple surgeries. His body mass index (BMI) was continuously low. Tissue injuries provoked inflammatory responses that contributed to metabolic disarrangement, meanwhile the presence of psychiatric disorder complicated the means of nutrition assessment and therapy needed to compensate for the high nutrition demand during the treatment period. Nutrition support should be assessed and adjusted continuously.

## Introduction

Burn injury, particularly that affect a large total body surface area (TBSA), could induce a hypermetabolic state characterized by accelerated protein catabolism and increased oxygen consumption [1]. On the other hand, nutrition intake often fell significantly. The presence of psychiatric comorbidity may worsen the nutrition status due to adverse medication effects, negative affective symptoms, and reduced nutrition intake [2]. Surgery, as one of the treatment options for burns, could also lead to tissue inflammation that corresponds with the extent of the surgical trauma. Moreover, the success of the surgery does not depend exclusively on surgical skills, but also the ability of the patient to carry the metabolic load [3]. We report the case of a severe burn patient with schizophrenia to explore various factors that play a role in nutrition problems in this patient.

## Patient and observation

**Figure 1:**
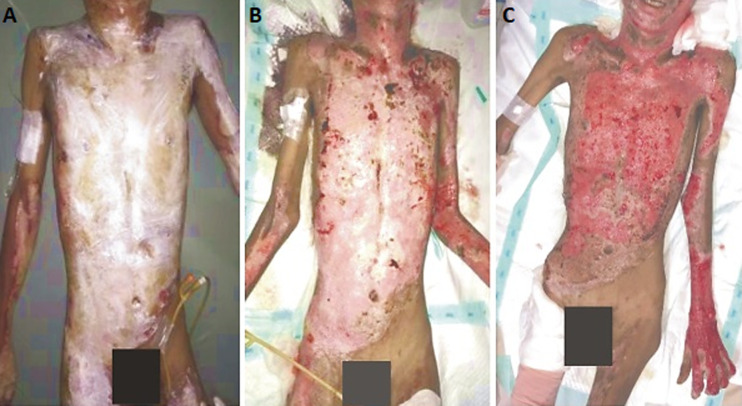
evaluation of clinical status and BMI: A) day 1, BMI 17.57 kg/m^2^; B) day 28, BMI 12.94 kg/m^2^; C) day 66, BMI 11.96 kg/m^2^

A 35-year-old mentally ill man was transferred from a local psychiatric hospital after attempted suicide by fire 3 days before admission. He acquired full- and partial-thickness injury in approximately 38% of his TBSA, including face and neck (5%), both upper extremities (8%), right thigh (4%), left thigh and leg (3%), and most of his anterior and posterior trunk (16%). The patient was hospitalized for 66 days and underwent five debridement surgeries. The psychiatric diagnosis was schizophrenia type ICD 10 F20.3, treated with Haloperidol 1.5 mg and Diazepam 5 mg daily. The patient was occasionally restrained due to rebellious and self-destruction behavior. The nutrition management of this patient was started after a week of hospitalization, which is considered late. In the initial nutrition assessment, the patient was 40 kg weight and 150 cm tall thus the body mass index (BMI) was 17,57 kg/m^2^ or underweight (Figure 1A). Gastrointestinal symptoms and signs were not found. Information regarding the patient´s dietary intake in the last two weeks and weight change within the last 6 months could not be obtained, but his mother claimed that the patient seemed to lose weight because he refused any food that had been offered to him; he only smoked and drank coffee. Albumin level was 3.6 gr/L with imbalanced electrolyte levels. Signs of fluid accumulation were not found. According to the subjective global assessment (SGA) tool, the patient belonged to group C (severely malnourished). Energy and protein were given starting from 30 kcal/kg, increasing gradually to 50 kcal/kg, and 2 gp/kg divided into oral nutrition and oral nutrition supplements (ONS) with a 50: 50 ratio. The patient was also given micronutrient supplementations, specifically vitamin A 100,000 units for the first week, vitamin C 100 mg 3 times a day, and zinc 20 mg once a day.

During the early weeks of hospitalization, the patient´s weight loss continued. At the end of the second week, his body weight was 36 kilograms while his serum albumin level was 2.7 gr/L. On the 28^th^ day, the patient´s body weight was 33 kilograms while his serum albumin level was 2.1 gr/L (Figure 1B). Energy and protein intake was maintained, but the ratio of oral intake and ONS was modified to 70: 30. During the treatment, the patient´s appetite was unstable. The patient was also hardly cooperative with enteral access. The patient often writhed in pain and became more aggressive. The patient did not respond well to analgesics and sedations. Partially parenteral nutrition (PN) enriched with branched-chain amino acid (BCAA) was administered to support protein needs. However, his BMI continued to decline, and on the 42^nd^ day, his body weight dropped to 32 kilograms with a serum albumin level of 2 gr/L. On the 66^th^ day, granulation tissues appeared on the wound bed. Considering the mental and nutrition status of the patient, it was decided not to continue with the skin graft surgery. The patient had lost 24% of body weight (BMI 11.96 kg/m^2^) and the serum albumin level was 2.5 gr/L (Figure 1C). The patient displayed severe muscle wasting and subcutaneous fat loss. However, the general and mental condition was relatively better. The patient was discharged from the hospital.

## Discussion

More than two-thirds of deaths among patients with schizophrenia were attributed to severe malnutrition and other diseases. A higher prevalence of underweight was found in a cohort of 23.116 adults with schizophrenia than in the general population in Japan (13.8% vs 7.9%). In Asia, it has been reported that the prevalence of underweight and undernutrition in chronically hospitalized patients with schizophrenia is substantially higher than that in the general population [2]. Schizophrenia may increase morbidity due to self-destructive behavior, changes in nutrition intake, sleep disturbance, and medications that influence the immune system. Haloperidol particularly antagonized the dopamine D2 receptor. Activation of this receptor generally inhibit food intake, reduces body weight, and enhance insulin secretion [4]. Metabolic derangements secondary to major burn injuries are difficult to manage. Immediately after a severe burn injury, plasma volume is depleted while insulin levels lowered oxygen consumption, body temperature, and overall metabolic rate. This “ebb” phase is followed by an evolving “flow” phase in weeks following injury. These post-burn metabolic-inflammatory disorders could last for years [1,3].

**Figure 2:**
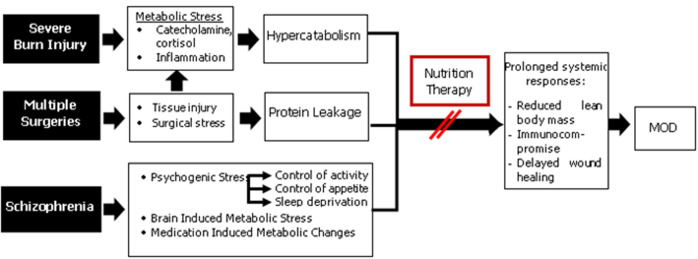
analysis of pathophysiology (MOD: multiple organ dysfunctions)

Elevated stress hormones and pro-inflammatory cytokines along with excessive mitochondrial-derived free radicals lead to an insulin receptor impairment that counteracts insulin anabolism. Those factors were assumed to play a role in stimulating muscle proteolysis, protein breakdown, and protein oxidation. The high rate of protein oxidation account for elevated energy expenditure in burn patients. This situation resulted in a persistent negative net protein balance, which likely contributed to the prolonged cachexia in burn victims [5]. Tissue trauma from surgery also accelerated the protein catabolism process that may cause further protein loss. Albumin has been identified as a reliable indicator of this phenomenon. Capillary leak due to the metabolic stress response probably represented the principal mechanism of postoperative albumin reduction [3,5]. In severely burned patients with comorbidities, the length of stay is often prolonged due to delayed wound healing, infections, multiple surgery requirements, and a long period of immobilization. Malnutrition is also associated with more severe conditions, such as multiple organ dysfunctions or death (Figure 2) [6].

**Nutrition assessment and support:** to achieve proper recovery, adequate metabolic responses are required. At the time of admission, factors related to the patient´s pre-burn history (including days post-burn, prior burn care, and any complicating injuries), pre-burn height and weight, and clinical appearance serve as the basis for the patient´s initial nutritional assessment [1,7]. However, the initial nutrition assessment was hardly done to the patient due to inadequate information sources and questionable validity. Therefore, the patient´s family played a big role in nutrition screening and evaluation. The current gold standard for measuring energy expenditure is indirect calorimetry. Unfortunately, it is not practical in a routine clinical setting. Various equations have been developed to estimate nutrition and calorie requirements for burn patients. Sugawara and colleagues suggested that the Harris-Benedict equation is the most appropriate to estimate resting energy expenditure (REE) and predicted energy expenditure (PEE) in schizophrenic patients with antipsychotic medications (Table 1) [2,4]. However, this formula could only act as a guide. Energy expenditure fluctuated after burn injury, and strictly following this formula could lead to underfeeding during periods of high energy utilization and overfeeding later during the recovery phase. Besides, changes in physical stress resulted from repeated surgery and inconsistent pattern of activity could affect energy expenditure [6-8].

**Table 1:** Harris-Benedict and associated stress factors used in the calculation of predicted energy expenditure (PEE)

PEE	
Male	PEE = 66 + (13,7 x wt) + (5 x ht) - (6 x age)
Female	PEE = 655 + (9.6 x wt) + (1.7 x ht) - (4.7 x age)
Stress/injury factor	
≤10% burn	1.0 to 1.1
10-25% burn	1.1 to 1.3
25-90% burn	1.2 to 1.7
Minor surgery	1.1 to 1.3
Major surgery	1.2 to 1.4
Multiple trauma	1.4 to 1.6
Activity factor	
Bed bound immobile	1,1
Bed bound mobile/sitting	1,15-1,2
Mobile on ward	1,25

Note: Weight (wt) is expressed in kilograms, height (ht) is expressed in centimeters, and age is expressed in years.

According to the American Burn Association practice guideline, enteral nutrition (EN) should begin as soon as possible. It is recommended to initiate EN within 24 hours of injury, and research indicated starting EN as early as 6 hours post-injury is safe, effective, and can reverse the detrimental effects of metabolic and hormonal shifts [8,9]. However, malnourished patients who are intolerant to EN and patients with postoperative complications that made them unable to have adequate oral/ enteral intake for at least 7 days should be considered to be given PN. The recent ASPEN guideline also recommended postoperative PN for patients who cannot meet their energy needs orally/ enterally within 5-7 days. Lastly, continuous nutrition status evaluation and therapy adjustment are very important [8,10].

## Conclusion

Many factors contribute to metabolic stress in burn patients with schizophrenia and malnutrition, making nutrition problems very complicated. Nutrition therapy needs to be initiated early and tuned individually. Changes in clinical status prompted a continuous reassessment of nutrient requirements. Collaboration between medical personnel and the patient´s family is needed to ensure comprehensive management.
